# The associations between changes in hepatic steatosis and heart failure and mortality: a nationwide cohort study

**DOI:** 10.1186/s12933-022-01725-z

**Published:** 2022-12-23

**Authors:** Jiyun Park, Gyuri Kim, Hasung Kim, Jungkuk Lee, Sang-Man Jin, Jae Hyeon Kim

**Affiliations:** 1 Department of Internal Medicine, CHA Bundang Medical Center, CHA University School of Medicine, Seongnam, 13496 Republic of Korea; 2 Sungkyunkwan University School of Medicine, Seoul, Republic of Korea; 3 Department of Medicine, Samsung Medical Center, Sungkyunkwan University School of Medicine, 81, Irwon-Ro, Gangnam-Gu, Seoul, 06351 Republic of Korea; 4 Data Science Team, Hanmi Pharm. Co. Ltd, Seoul, Republic of Korea; 5 Department of Clinical Research Design and Evaluation, Samsung Advanced Institute for Health Sciences and Technology, Seoul, Republic of Korea

**Keywords:** Fatty liver index, Heart failure, Mortality, Non-alcoholic fatty liver disease

## Abstract

**Background:**

Non-alcoholic fatty liver disease (NAFLD) is a well-known risk factor for cardiovascular (CV) disease (CVD) and mortality. However, whether the progression or regression of NAFLD can increase or decrease the risk of heart failure (HF) and mortality has not been fully evaluated. We investigated the association between changes in hepatic steatosis and the risks of incident HF (iHF), hospitalization for HF (hHF), and mortality including CV- or liver-related mortality.

**Methods:**

Using a database from the National Health Insurance Service in Korea from January 2009 to December 2012, we analyzed 240,301 individuals who underwent health check-ups at least twice in two years. Hepatic steatosis was assessed using the fatty liver index (FLI), with an FLI ≥ 60 considered to indicate the presence of hepatic steatosis. According to FLI changes, participants were divided into four groups. Hazard ratios (HRs) and 95% confidence intervals (CIs) were estimated using multivariable Cox proportional hazards regression models.

**Results:**

Persistent hepatic steatosis increased the risk of iHF, hHF, and mortality including CV- and liver-related mortality compared with the group that never had steatosis (all P < 0.05). Incident hepatic steatosis was associated with increased risk for iHF and mortality including CV- or liver-related mortality (all P < 0.05). Compared with persistent steatosis, regression of hepatic steatosis was associated with decreased risk for iHF, hHF, and liver-related mortality (iHF, HR [95% CI], 0.800 [0.691–0.925]; hHF, 0.645 [0.514–0.810]; liver-related mortality, 0.434 [0.223–0.846]).

**Conclusions:**

FLI changes were associated with increased or decreased risk of HF outcomes and mortality.

**Supplementary Information:**

The online version contains supplementary material available at 10.1186/s12933-022-01725-z.

## Background

Non-alcoholic fatty liver disease (NAFLD) is defined as excessive hepatic fat accumulation confirmed by imaging or histology in the absence of a secondary cause of hepatic steatosis such as excessive alcohol consumption [1, 2]. NAFLD represents a spectrum of disease ranging from simple steatosis to steatohepatitis, advanced fibrosis, and cirrhosis [3]. Hepatic fat accumulation induces insulin resistance, which impairs hepatic metabolism and induces inflammation in the liver [4], and those processes are key in explaining the associations among NAFLD, metabolic disease, and cardiovascular disease (CVD) [5]. NAFLD has traditionally been regarded as hepatic manifestation of metabolic syndrome; however, bidirectional relationship between NAFLD and metabolic syndrome, diabetes, and CVD respectively, has recently been highlighted [5–8]. Those studies reported that NAFLD plays an important role in the development of metabolic syndrome, diabetes, and CVD independent of other metabolic and cardiovascular risk factors.

According to reports evaluating the natural course of NAFLD in the general population, NAFLD incidence is 18.5–36.7% and established NAFLD can persist or improve, with a reported remission rate of 24.6–46.1% during 6–8 years [9–11]. In shorter 1.1-year follow-up study in Japan, incidence rate was 10% and remission rate was 16% [12]. Several studies have reported that resolution of a fatty liver is associated with a decrease in incident diabetes and improved lipid profiles but not with a decrease in incident hypertension [13–15]. However, it has not been determined whether incident, sustained, or remitted NAFLD is associated with CVD, especially heart failure (HF) which is one of the most important global health problems.

The fatty liver index (FLI) is a useful marker of fatty liver and it was validated in predicting fatty liver confirmed by ultrasound [16, 17]. It was previously reported that an increased FLI is associated with an increased risk of CVD and related mortality [18, 19]. In our previous study, we reported that the FLI was associated with incident HF (iHF), hospitalized HF (hHF), and related mortality in both the general population and patients with pre-existing HF [20]. In this study, we evaluated the association between changes in FLI over two years and the risk of iHF, hHF, and cardiovascular- or liver-related mortality in a general population.

## Methods

### Data source from the national health insurance service

We used a database from the National Health Insurance Service (NHIS) in Korea from January 2009 to December 2012. The NHIS covers 100% of the Korean population, providing medical services and health screenings and collecting necessary information on patient demographics and medical utilization/transactions in a series of databases [21, 22]. We used claims and health check-up data. The claims database contains the principle diagnosis and first additional diagnosis in the form of International Classification of Disease 10th revision (ICD-10) codes, number of days on which patients visited a medical facility, hospitalizations, and prescriptions. The health check-up database contains the responses to questionnaires about medical history, current medications, and lifestyle habits; anthropometric measurements; and laboratory test results [22]. Information about the cause of death, which was classified using the Korean Standard Classification of Disease and Cause of Death, was provided by the Korean National Statistical Office. This study was approved by the Institutional Review Board of Samsung Medical Center (approval no. SMC 2019-11-051), Seoul, Republic of Korea, who granted an exemption to the need for informed consent because all data provided to the researchers were de-identified.

### Study population

**Fig. 1:**
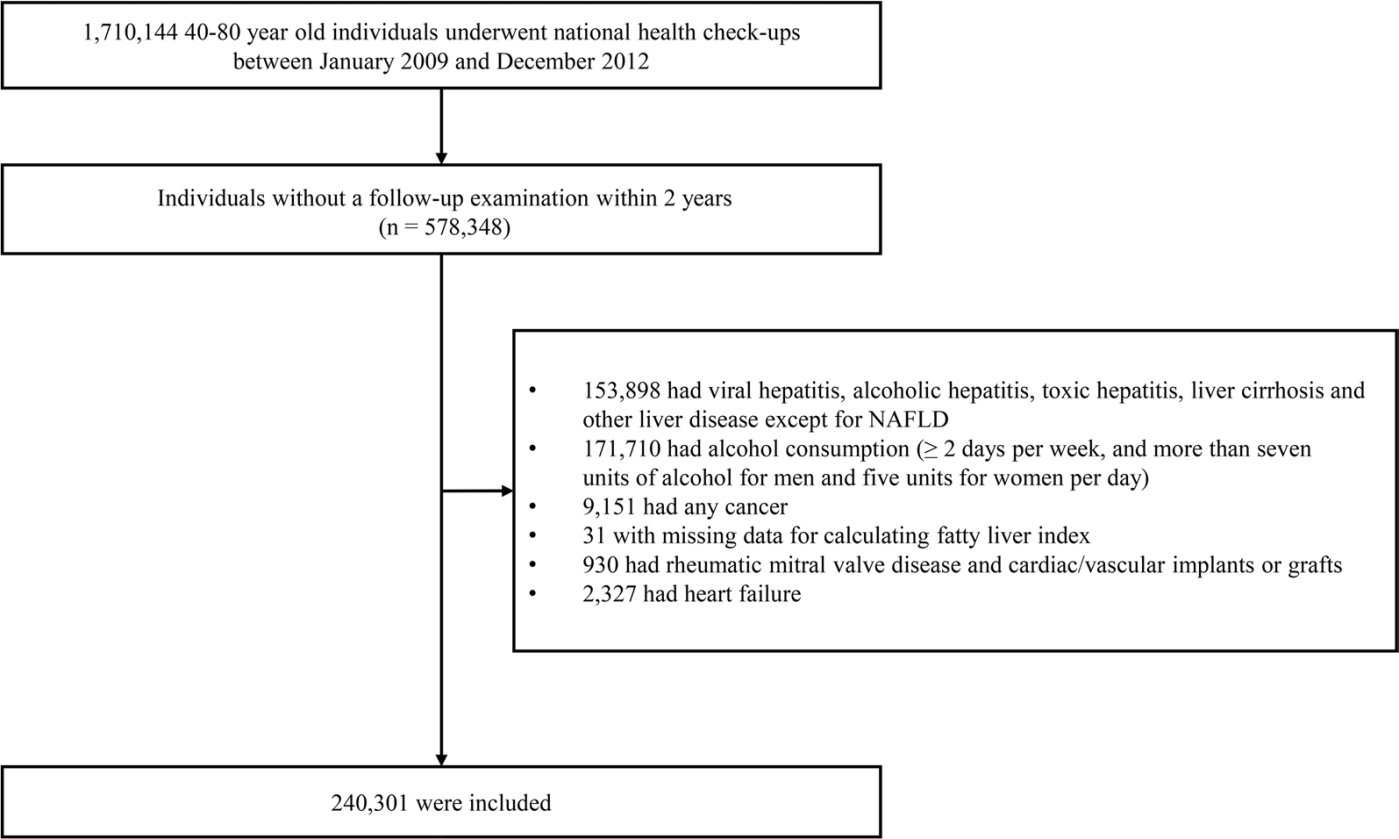
Flow diagram of the study population. NAFLD, Non-alcoholic fatty liver disease

We requested the data of individuals aged 40–80 years the most common group of NAFLD [23], who underwent regular health check-ups at least between January 2009 and December 2012 from NHIS. Due to the large amount of data, we could use the data on subjects who were stratified by age and sex and extracted 10% (n = 1,710,144). Among them, we selected individuals who received at least two health check-ups within 2 years (n = 578,348). We excluded 153,898 individuals who had hepatitis or a liver disease other than NAFLD; 171,170 individuals who consumed alcohol at least two days per week or consumed more than seven units of alcohol for males or five units for females per day (daily unit × number of times per week ≥ 14 in men and ≥ 10 in women) [19, 20]; 9,151 individuals who had cancer of any type; 31 individuals who did not have data for calculating the FLI; 930 individuals who had rheumatic mitral valve disease or cardiac/vascular implants or grafts; and 2,327 individuals who had pre-existing HF (Fig. 1). In that way, we included 240,301 individuals in this analysis.

### Measurements of clinical and biochemical parameters

The information acquired from questionnaires was age, sex, smoking, alcohol consumption, regular exercise, and income. Regular exercise was determined as high-intensity physical activity for at least 20 min at least three times per week or moderate-intensity physical activity performed for at least 30 min at least 5 times per week [24, 25]. Income was divided by quartile based on monthly income. Body mass index (BMI) was calculated as BMI = body weight (kg)/height^2^ (m^2^). Waist circumference (WC) was measured at the midpoint between the lower costal margin and the iliac crest. Blood samples were collected after overnight fasting and analyzed for fasting glucose, total cholesterol, triglycerides, low-density lipoprotein cholesterol, high-density lipoprotein cholesterol, aspartate aminotransferase (AST), alanine aminotransferase (ALT), and γ-glutamyl transferase (GGT). The estimated glomerular filtration rate (eGFR) was calculated according to the Chronic Kidney Disease Epidemiology Collaboration equation. The definitions of hypertension, diabetes, dyslipidemia, and metabolic syndrome were based on previous studies [20, 26].

### Definitions of hepatic steatosis and steatosis status changes

Hepatic steatosis was defined according to the well-validated FLI [16, 17], which was calculated as (e^0.95 × log^_e_^(triglyceride) + 0.139 × BMI + 0.718 × log^_e_^(ggt) + 0.053 × WC − 15.745^)/(1 + e^0.95 × log^_e_^(triglyceride) + 0.139 × BMI + 0.718 × log^_e_^(ggt) + 0.053 × WC − 15.745^) × 100. Individuals with FLI < 60 were considered to have a low probability of hepatic steatosis, and those with FLI ≥ 60 were considered to have a high probability of hepatic steatosis [16, 27]. In addition, using FLI results from two examinations in two years, we divided the participants into four groups: (1) non-NAFLD, FLI < 60 at the first exam and FLI < 60 at the second exam, (2) regressed NAFLD, FLI ≥ 60 at the first exam and FLI < 60 at the second exam, (3) incident NALFD, FLI < 60 at the first exam and FLI ≥ 60 at the second exam, (4) persistent NAFLD, FLI ≥ 60 at the first exam and FLI ≥ 60 at the second exam.

### Outcomes

iHF was defined as a first hospital visit of at least two outpatient hospital visits or a first event of hHF with the ICD-10 disease code I50 [20]. iHF included both primary and secondary diagnoses. hHF was defined as a first hospitalization with a primary diagnosis of ICD-10 disease code I50 [20, 28]. CV mortality was defined as death caused by ischemic heart disease, HF, cerebrovascular disease, or cardiac arrest, as shown by the relevant ICD-10 codes (I20–I25, I50, I60-69, G45, I46) [29]. Liver-related mortality was caused by alcoholic liver disease, liver cirrhosis, unclassified chronic hepatitis, liver failure, or hepatocellular carcinoma (HCC), as shown by the relevant ICD-10 codes (K70, K72–76, C22) [29, 30].

### Statistical analysis

Continuous variables are expressed as the means ± standard deviations. Categorical data are expressed as numbers with percentages. Group comparisons were performed using a one-way analysis of variance for continuous variables and chi-square testing for categorical variables. Multivariable Cox proportional hazards regression models were used to evaluate hazard ratios (HRs) and 95% confidence intervals (95% CIs) for HF outcomes and mortality during follow up, including CV mortality. The covariates for adjustment were (1) Model 1, crude; (2) Model 2, age, sex, and body weight; (3) Model 3, covariates in Model 2 + alcohol consumption, smoking, regular exercise, and income status; (4) Model 4, covariates in Model 3 + hypertension, diabetes, dyslipidemia, and eGFR. Since the FLI can be changed during subsequent periods after the first two years, subjects whose FLI categories were subsequently changed were censored for sensitivity analysis. The p-values for interaction were evaluated through an analysis stratified by age (< 60 years vs. ≥ 60 years) [31] and BMI (< 25 kg/m2 vs. ≥ 25 kg/m2) [20, 32]. A p-value less than 0.05 was considered statistically significant. Statistical analyses were performed using the SAS software program (version 9.4; SAS Institute, Cary, NC, USA).

## Results

### Baseline characteristics of the study population

Among the 240,301 subjects included in our study, 206,538 (85.95%) were in the non-NAFLD group, 9212 (3.83%) were in the regressed NAFLD group, 9641 (4.01%) were in the incident NAFLD group, and 14,910 (6.20%) were in the persistent NAFLD group. The baseline characteristics of the study population according to FLI changes are presented in Table 1, which shows that the mean values of BMI, WC, systolic blood pressure, fasting glucose, AST, ALT, GGT, and triglycerides all increased through the groups in the order of non-NAFLD, regressed NAFLD, incident NAFLD, and persistent NAFLD. The proportion of the subjects with dyslipidemia, metabolic syndrome and eGFR ≤ 60 was increased in order of the groups mentioned above. Hypertension and diabetes occurred most frequently in the persistent NAFLD group and were least frequent in the non-NAFLD group.

**Table 1:** Baseline characteristics of the study population according to FLI change (n = 240,301)

	Non-NAFLD	Regressed NAFLD	Incident NAFLD	Persistent NAFLD	p-value
n (%)	206,538 (85.95)	9212 (3.83)	9641 (4.01)	14,910 (6.20)	< 0.001
Age (years)	52.69 ± 9.42	53.62 ± 9.52	52.35 ± 9.16	51.76 ± 8.93	< 0.001
Males	103,081 (49.91)	7444 (80.81)	7614 (78.98)	12,797 (85.83)	< 0.001
Income level, lowest 25%	47,125 (22.82)	1821 (19.77)	1966 (20.39)	2843 (19.07)	< 0.001
Current smoker	38,815 (18.79)	3,049 (33.10)	3,075 (31.90)	5855 (39.27)	< 0.001
Regular exercise	51,489 (24.93)	2,389 (25.93)	2,012 (20.87)	3130 (20.99)	< 0.001
Body weight (kg)	61.47 ± 9.13	71.88 ± 8.17	74.60 ± 8.63	79.85 ± 9.73	< 0.001
BMI (kg/m^2^)	23.22 ± 2.53	25.97 ± 2.29	26.94 ± 2.57	28.32 ± 2.90	< 0.001
Waist circumference (cm)	78.78 ± 7.50	86.66 ± 5.58	90.39 ± 5.79	93.28 ± 6.68	< 0.001
In males	81.66 ± 6.34	86.79 ± 5.38	90.27 ± 5.52	92.98 ± 6.50	< 0.001
In females	75.90 ± 7.45	86.11 ± 6.33	90.83 ± 6.66	95.08 ± 7.48	< 0.001
SBP (mmHg)	121.00 ± 14.53	126.81 ± 14.26	128.48 ± 14.27	130.17 ± 14.50	< 0.001
DBP (mmHg)	75.60 ± 9.73	79.20 ± 9.64	80.81 ± 9.70	81.97 ± 10.07	< 0.001
Fasting plasma glucose (mg/dl)	96.60 ± 19.42	104.82 ± 27.93	105.64 ± 26.12	110.49 ± 32.25	< 0.001
AST (IU/L)	23.39 ± 11.74	25.54 ± 10.45	32.52 ± 48.61	31.88 ± 20.67	< 0.001
ALT (IU/L)	21.08 ± 15.13	27.06 ± 14.24	39.13 ± 40.42	39.42 ± 26.00	< 0.001
GGT (IU/L)	26.40 ± 21.94	44.92 ± 35.08	73.94 ± 75.99	82.80 ± 79.60	< 0.001
Total cholesterol (mg/dl)	197.32 ± 34.79	200.70 ± 37.44	212.74 ± 37.97	211.44 ± 40.01	< 0.001
Triglycerides (mg/dl)	112.53 ± 61.01	151.20 ± 70.42	246.09 ± 39.52	249.82 ± 54.52	< 0.001
HDL-C (mg/dl)	56.58 ± 16.04	50.23 ± 14.77	48.89 ± 15.07	47.50 ± 14.33	< 0.001
LDL-C (mg/dl)	118.63 ± 36.83	120.87 ± 41.85	117.42 ± 42.92	117.28 ± 49.05	< 0.001
eGFR < 60 (ml/min/1.73m^2^)	40,258 (19.49)	2908 (31.57)	3051 (31.65)	4974 (33.36)	< 0.001
Comorbidities					
Hypertension	52,227 (25.29)	4112 (44.64)	4205 (43.62)	7565 (50.74)	< 0.001
Dyslipidemia	43,821 (21.22)	3316 (36.00)	3532 (36.64)	6047 (40.56)	< 0.001
Diabetes mellitus	14,114 (6.83)	1623 (17.62)	1419 (14.72)	3118 (20.91)	< 0.001
Metabolic syndrome^a^	51,166 (24.77)	4786 (51.95)	7247 (75.17)	11,976 (80.32)	< 0.001

Continuous variables are expressed as means ± standard deviations. Categorical data are presented as frequencies and percentages

*AST* alanine aminotransferase, *ALT* aspartate aminotransferase, *BMI* body mass index, *DBP* diastolic blood pressure, *eGFR* estimated glomerular filtration rate, *FLI* fatty liver index, *GGT* gamma-glutamyl transferase, *HDL-C* high-density lipoprotein cholesterol, *LDL-C* low-density lipoprotein cholesterol, *SBP* systolic blood pressure

^a^Metabolic syndrome was defined as three or more of the following five risk factors: Waist circumference ≥ 90 in males and ≥ 80 in females, triglycerides ≥ 150 mg/dl, HDL < 40 in males and < 50 in females, blood pressure ≥ 130/ ≥ 85, and fasting glucose ≥ 100 mg/dl

### The associations between FLI changes and iHF and hHF

During a median follow-up period of 6.7 years, 6,186 (2.57%) subjects developed iHF, and 2579 (1.07%) developed hHF. Table 2 shows the crude and multivariable adjusted HRs and CIs for iHF and hHF according to FLI changes. Compared with the non-NAFLD group, the other three groups had a significantly increased risk of iHF and hHF in the crude model. In the fully adjusted model 4, the HR of the regressed NAFLD group was attenuated and lost its significant association with iHF (adjusted HR (aHR), 1.049; 95% CI 0.935–1.178). However, the significant association between FLI changes and iHF remained in the incident and persistent NAFLD groups (FLI < 60/FLI ≥ 60, 1.150 [1.024–1.292]; FLI ≥ 60/FLI ≥ 60, 1.316 [1.194–1.450]). Only the persistent NAFLD group had a significant association with hHF in the fully adjusted model (FLI ≥ 60/FLI ≥ 60, 1.666 [1.434–1.935]). When we further compared the regressed NAFLD group with the persistent NAFLD group for development of HF or mortality during follow up, the HRs for iHF and hHF were significantly lower in the regressed NAFLD group (iHF, 0.800 [0.691–0.925]; hHF 0.645 [0.514–0.810]).

**Table 2:** Risks of incident heart failure and hospitalization for heart failure according to FLI changes

	Events	Model 1			Model 2			Model 3			Model 4		
		HR	95% CI	P value	HR	95% CI	P value	HR	95% CI	P value	HR	95% CI	P value
iHF													
Non-NAFLD	4902	Ref.			Ref.			Ref.			Ref.		
Regressed NAFLD	326	1.507	1.348–1.686	< 0.001	1.237	1.102–1.388	< 0.001	1.219	1.087–1.368	< 0.001	1.049	0.935–1.178	0.414
Incident NAFLD	332	1.478	1.322–1.651	< 0.001	1.326	1.181–1.489	< 0.001	1.307	1.164–1.469	< 0.001	1.150	1.024–1.292	0.018
Persistent NAFLD	626	1.827	1.681–1.986	< 0.001	1.619	1.471–1.782	< 0.001	1.575	1.430–1.736	< 0.001	1.316	1.194–1.450	< 0.001
Persistent NAFLD	626	Ref.			Ref.			Ref.			Ref.		
Regressed NAFLD	326	0.825	0.722–0.943	0.005	0.753	0.652–0.870	< 0.001	0.752	0.650–0.869	< 0.001	0.800	0.691–0.925	0.003
hHF													
Non-NAFLD	2,067	Ref.			Ref.			Ref.			Ref.		
Regressed NAFLD	129	1.408	1.179–1.682	< 0.001	1.285	1.071–1.542	0.007	1.249	1.041–1.499	0.017	1.101	0.916–1.322	0.305
Incident NAFLD	120	1.264	1.052–1.520	0.013	1.328	1.098–1.608	0.004	1.289	1.065–1.561	0.009	1.162	0.959–1.407	0.125
Persistent NAFLD	263	1.822	1.603–2.072	< 0.001	2.048	1.767–2.373	< 0.001	1.939	1.671–2.251	< 0.001	1.666	1.434–1.935	< 0.001
Persistent NAFLD	263	Ref.			Ref.			Ref.			Ref.		
Regressed NAFLD	129	0.773	0.626–0.954	0.017	0.607	0.484–0.760	< 0.001	0.616	0.491–0.773	< 0.001	0.645	0.514–0.810	< 0.001

*CI* confidence interval, *HR* hazard ratio, *iHF* incident heart failure, *hHF* hospitalized heart failure

Model 1: Crude

Model 2: Age, sex, and body weight

Model 3: Model 2 + alcohol consumption, smoking, regular exercise, and income status

Model 4: Model 3 + hypertension, diabetes mellitus, dyslipidemia, and estimated glomerular filtration rate

### The associations between FLI changes and all-cause, CV-, and liver-related mortality

Within our study population, 4,756 (1.98%) subjects died from any cause, including 673 (0.28%) of CVD-related death and 217 (0.09%) of liver-related deaths. Table 3 shows the crude and multivariable adjusted HRs and CIs for mortality during follow up according to FLI changes. Compared with the non-NAFLD group, the risk of all-cause and liver-related death during follow up increased through the groups in the order of regressed NAFLD, incident NAFLD, and persistent NAFLD in the fully adjusted model (all p < 0.05). The incident and persistent NAFLD groups were also associated with CV mortality in the fully adjusted model (FLI < 60/FLI ≥ 60, 1.673 [1.199–2.333]; FLI ≥ 60/FLI ≥ 60, 1.421 [1.028–1.963]). Compared with the persistent NAFLD group, the regressed NAFLD group was associated with a decreased risk of liver-related mortality but not with a decreased risk of CVD-related or all-cause mortality during follow up (liver-related mortality, 0.434 [0.223–0.846]).

**Table 3:** Risks of all-cause mortality, cardiovascular mortality, and liver-related mortality during follow up according to FLI changes

	Events	Model 1			Model 2			Model 3			Model 4		
		HR	95% CI	P value	HR	95% CI	P value	HR	95% CI	P value	HR	95% CI	P value
All-cause mortality													
Non-NAFLD	3,915	Ref.			Ref.			Ref.			Ref.		
Regressed NAFLD	257	1.477	1.302–1.676	< 0.001	1.490	1.309–1.696	< 0.001	1.415	1.242–1.611	< 0.001	1.320	1.158–1.504	< 0.001
Incident NAFLD	225	1.246	1.089–1.425	0.001	1.550	1.349–1.781	< 0.001	1.453	1.264–1.671	< 0.001	1.378	1.198–1.585	< 0.001
Persistent NAFLD	359	1.300	1.167–1.448	< 0.001	1.886	1.674–2.125	< 0.001	1.710	1.516–1.929	< 0.001	1.569	1.390–1.771	< 0.001
Persistent NAFLD	359	Ref.			Ref.			Ref.			Ref.		
Regressed NAFLD	257	1.137	0.968–1.334	0.112	0.825	0.695–0.979	0.003	0.862	0.725–1.024	0.091	0.879	0.739–1.045	0.145
CV mortality													
Non-NAFLD	549	Ref.			Ref.			Ref.			Ref.		
Regressed NAFLD	33	1.353	0.952–1.922	0.092	1.319	0.921–1.891	0.131	1.255	0.875–1.799	0.217	1.115	0.777–1.600	0.555
Incident NAFLD	41	1.619	1.179–2.223	0.003	1.958	1.406–2.726	< 0.001	1.848	1.325–2.578	< 0.001	1.673	1.199–2.333	0.003
Persistent NAFLD	50	1.291	0.966–1.724	0.084	1.807	1.313–2.487	< 0.001	1.628	1.180–2.246	0.003	1.421	1.028–1.963	0.033
Persistent NAFLD	50	Ref.			Ref			Ref.			Ref.		
Regressed NAFLD	33	1.045	0.673–1.622	0.845	0.837	0.521–1.345	0.462	0.846	0.525–1.362	0.492	0.859	0.533–1.386	0.534
Liver-related mortality													
Non-NAFLD	151	Ref.			Ref.			Ref.			Ref.		
Regressed NAFLD	14	2.086	1.207–3.608	0.009	2.279	1.295–4.010	0.004	1.255	0.875–1.799	0.217	1.986	1.123–3.511	0.018
Incident NAFLD	20	2.868	1.799–4.573	< 0.001	3.855	2.346–6.335	< 0.001	1.848	1.325–2.578	< 0.001	3.359	2.033–5.550	< 0.001
Persistent NAFLD	32	2.995	2.046–4.386	< 0.001	4.833	3.085–7.571	< 0.001	1.628	1.180–2.246	0.003	3.887	2.446–6.176	< 0.001
Persistent NAFLD	32	Ref.			Ref.			Ref.			Ref.		
Regressed NAFLD	14	0.698	0.373–1.308	0.262	0.421	0.218–0.814	0.010	0.437	0.225–0.850	0.015	0.434	0.223–0.846	0.014

*CI* confidence interval, *CV* cardiovascular, *HR* hazard ratio

Model 1: Crude

Model 2: Age, sex, and body weight

Model 3: Model 2 + alcohol consumption, smoking, regular exercise, and income status

Model 4: Model 3 + hypertension, diabetes mellitus, dyslipidemia, and estimated glomerular filtration rate

### Sensitivity analysis

After the first two years of FLI change, 33,736 of 240,301 individuals showed changed FLI categories in subsequent follow-up periods (Changes in FLI categories in 3rd exam, 17,445; in 4th exam, 8376; in 5th exam, 4307; in 6th exam, 1863; in 7th exam, 1063; 8th exam, 601; 9th exam, 81). Median follow-up period was 5.9 years in sensitivity analysis, the results is presented in Additional file 1: Table S1. The results of sensitivity analysis were similar in HR and statistical significance to the results of the entire population.

### Subgroup analyses

**Fig. 2:**
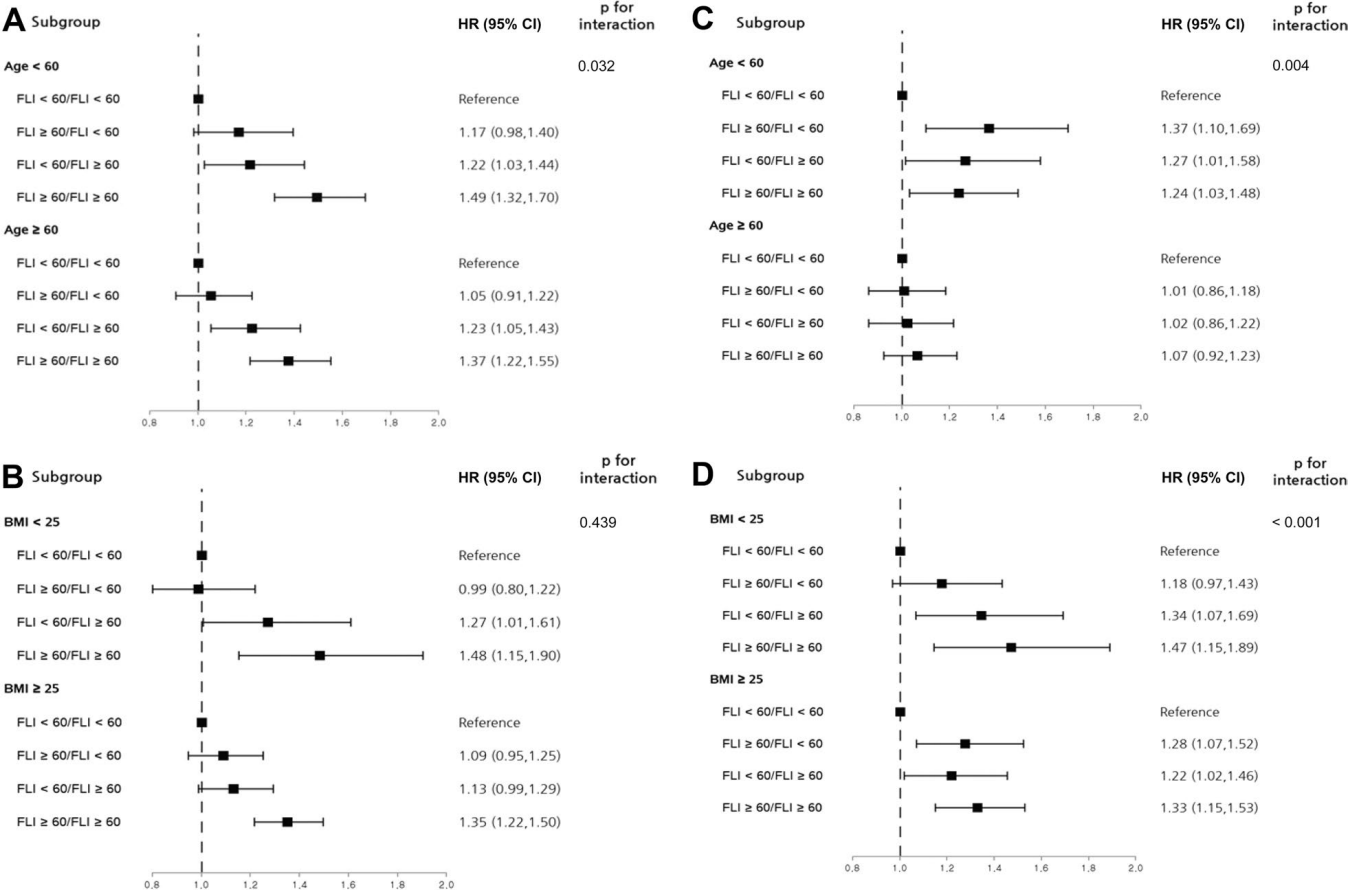
Subgroup analyses stratified by age and body mass index. **A**, **B**: Risk for incident heart failure according to change in fatty liver index **C**, **D**: Risk for mortality according to change in fatty liver index. *BMI* body mass index; *CI* confidence interval *FLI*, fatty liver index, *HR* hazard ratio

In subgroup analyses stratified by age and BMI and using the non-NAFLD group as the reference, iHF showed a tendency to increase through the groups in the order of regressed NAFLD, incident NAFLD, and persistent NAFLD (Fig. 2, A and B). The association between FLI changes and all-cause death during follow up remained significant only in subjects younger than 60 years, but FLI changes were associated with all-cause mortality during follow up regardless of BMI (Fig. 2C and D).

## Discussion

This is the first study to evaluate the association between changes in FLI over two years and iHF, hHF, and all-cause mortality during follow up (including CVD- and liver-related mortality) in a large, nationwide, population-based cohort. Compared with the non-NAFLD group, who maintained an FLI less than 60 for two years, the incident NAFLD group, whose FLI changed from less than 60 to 60 or more, and the persistent NAFLD group, who maintained an FLI of 60 or more for two years, had an increased risk of developing iHF. For hHF, the persistent NAFLD group had an increased risk compared with the non-NAFLD group. All-cause and liver-related mortality during follow up increased through the groups in the order of regressed NAFLD, incident NAFLD, and persistent NAFLD compared with the non-NAFLD group. The incident NAFLD and persistent NAFLD groups were associated with a higher risk of CVD-related mortality than the non-NAFLD group. Furthermore, compared with the persistent NAFLD group, iHF, hHF, and liver-related mortality occurred less frequently in the regressed NAFLD group. To the best of our knowledge, this is the first study to establish that FLI changes are significantly associated with development of iHF and hHF and the two most common causes of death in NAFLD patients.

NAFLD has been gathering attention as an important risk factor for development of CVD and related mortality independent of traditional risk factors. The precise mechanism explaining that association remains unknown, but insulin resistance, inflammation, endothelial dysfunction, oxidative stress, and intestinal dysbiosis, all of which affect myocardial or vascular structure directly or indirectly, are considered to play crucial roles [33–36]. Much evidence links NAFLD with CVD outcomes, but NAFLD is a state of continuously changing processes, so there is limited value in predicting CVD results from any particular state at a single point in time. A few previous reports considered changes in fatty liver status and metabolic outcomes. Those studies reported that resolution of fatty liver confirmed by ultrasound was associated with a decreased incidence of diabetes and improved lipid profiles [9, 37]. In addition, one study reported that FLI scores that were repeatedly elevated for up to four times were associated with an increased risk of myocardial infarction (MI), stroke, and mortality [38]. In addition, when the first and last exams in a previous study were compared, the incident NAFLD group showed a higher risk of CVD and mortality during follow up than did the group without NAFLD, and the group with improved NAFLD had lower risk of CVD and all-cause mortality during follow up than did the persistent NAFLD group [38]. Consistent with that previous result, our study additionally showed an association between FLI change and not only MI or stroke, but also development and progression of HF and CVD-related mortality, the most common cause of death in patients with NAFLD [39].

Although FLI is not a direct measurement for hepatic steatosis, FLI is a well-validated, non-invasive diagnosis of patients at risk of NAFLD. The area under receiving operating characteristic curves (AUROC) of FLI was 0.84 for hepatic steatosis in ultrasound [16]. In addition, several studies showed that FLI had reasonable accuracy for detecting hepatic steatosis in Asian [40–42]. Our study results show that ‘changes’ of FLI also can be used for risk stratification for HF in general population. HF is most important public health problem worldwide because it is common causes of morbidity and mortality [43]. Because there is not specific treatment for HF, its risk factors should be monitored closely. NAFLD is one of the well-known risk factors for development and progression of HF [20, 36, 44]. In addition to established bidirectional relationship between NAFLD and HF with reduced ejection fraction (HFrEF), recently the association between HF with preserved ejection fraction (HFpEF) was also highlighted [43, 45, 46]. These evidences mean that NAFLD affect subclinical changes of left ventricular structures and function and determine the onset and phenotype of HF [47]. However, whether changes in NAFLD status can affect HF outcomes was not evaluated yet. Since it is difficult to serially follow biopsy or imaging diagnostic methods, our study result is meaningful because it provides important clues for future prospective studies about the relationship between regression or progression of hepatic steatosis and CVD and related mortality.

In this study, we also evaluated death from NAFLD-related liver complications, such as liver cirrhosis and HCC. The risk factors for development of liver cirrhosis and HCC in NAFLD are evaluated in several previous studies [48, 49]. The improvements of risk factors such as body weight loss or reduction in alcohol intake have been reported to regression of hepatic steatosis or fibrosis [9, 10], but few studies have shown that these improvements directly reduce liver cirrhosis or HCC. Our study showed persistent increased hepatic steatosis increased risk of liver-related mortality and regression of hepatic steatosis decreased liver-related mortality. These findings proposed that the fact NAFLD should be considered in changing process and its prevention and treatment is important.

Treatment strategies for NAFLD involve identifying and treating related metabolic conditions, such as obesity, diabetes, and hypertension, and improving insulin resistance through weight loss, exercise, or pharmacological treatment [50]. Lifestyle modifications such as a hypocaloric diet, exercise, and weight loss have also been recommended to treat NAFLD in the general population because they improve hepatic steatosis [1]. This study emphasizes the importance of improving and preventing the progression of hepatic steatosis by showing that changes in FLI affect HF outcomes, including mortality. It also provides evidence supporting the need for lifestyle modifications to improve hepatic steatosis and prevent HF and mortality in the general population. Also with regard to drugs, our study supports that glucagon-like peptide 1 receptor agonists (GLP-1RAs) and sodium-glucose cotransporter-2 (SGLT-2) inhibitors may be attractive therapeutic options for patients with NAFLD and HF. Because GLP-1 RAs and SGLT-2 inhibitors significantly reduced hHF and improved histologic resolution of steatohepatitis [36]. Potential mechanisms of GLP-1 RAs and SGLT-2 inhibitors for NAFLD and HF are explained by weight reduction, improvement of insulin resistance, reduction of metabolic dysfunction, improvement of lipotoxic effects, and inflammation [51, 52]. However, during our study period from 2009 to 2012, SGLT-2 inhibitor was not released and the use of GLP-RA was almost insignificant in Korea [53], therefore we could not evaluate the effects of these drugs on HF and CV mortality. Future studies on the effect of the use of SGLT-2 inhibitors or GLP-1 RAs in patients with NAFLD on HF outcome are warranted.

## Strengths and limitations

The strength of this study is that it is a large population-based longitudinal study evaluating the association between changes in FLI, a readily available surrogate marker for NAFLD, over 2 years and HF outcomes, including mortality. In addition, we are the first to analyze the causes of death most likely to be related to NAFLD in terms of FLI changes in a general population. However, this study also has several limitations. First, our NAFLD diagnosis is based on FLI rather than imaging or biopsy. The Korean NHIS database we used does not include ultrasound data; therefore, we could not match FLI scores with the extent of hepatic steatosis based on ultrasound. However, many studies have validated FLI as a marker for hepatic steatosis [16, 17]. Second, we diagnosed HF using diagnostic codes in the NHIS claims dataset. We could not evaluate symptoms, signs, or echocardiography, so misdiagnoses could be included. However, we tried to reduce the effects of misdiagnosis using the strictest possible criteria for iHF and hHF, following the lead of previous studies [20]. Third, we adjusted many risk factors for outcomes such as age, sex, body weight, and other metabolic factors, however, we could not adjust for unmeasured confounding factors such as NAFLD severity, inflammation markers, and degree of insulin.

In summary, the group with persistent hepatic steatosis for 2 years, as assessed by FLI, was associated with an increased risk of iHF, hHF, and mortality during follow up compared with the group never diagnosed with steatosis. Incident hepatic steatosis increased the risk of iHF and mortality during follow up. Furthermore, compared with persistent hepatic steatosis, regressed hepatic steatosis decreased the risk for iHF, hHF, and liver-related mortality. This study examined hepatic steatosis in terms of dynamic changes and could help physicians to identify patients at high risk of HF or mortality so that they can actively provide education about lifestyle interventions that can prevent or ameliorate hepatic steatosis in those patients.

## Supplementary Information

**Additional file 1: Table S1.** Sensitivity analysis.

## Data Availability

The data that support the findings of this study are available form Korean National Health Insurance Service (KNHIS), but restrictions apply to their availability. However, data are available from the authors upon reasonable request and with permission from the KNHIS.

## Abbreviations

CIs: Confidence intervals
CVD: Cardiovascular disease
FLI: Fatty liver index
HF: Heart failure
hHF: Hospitalized heart failure
HRs: Hazard ratios
NAFLD: Non-alcoholic fatty liver disease
iHF: Incident heart failure
