# Supplementary material for: The associations between changes in hepatic steatosis and heart failure and mortality: a nationwide cohort study
Source: Cardiovasc Diabetol. 2022 Dec 23;21:287. doi: 10.1186/s12933-022-01725-z (PMC9789584; doi:10.1186/s12933-022-01725-z)
Supplement: Supplementary file 1 — Additional file 1: Table S1. Sensitivity analysis. [file 12933_2022_1725_MOESM1_ESM.docx]

**The Associations Between Changes in Hepatic Steatosis and Heart Failure and Mortality: A Nationwide Cohort Study**

Jiyun Park^1,2,^**^†^**; Gyuri Kim^3,^**^†^**; Hasung Kim^4^; Jungkuk Lee^4^; Sang-Man Jin^3^; Jae Hyeon Kim^3,5,*^

^1^Department of Internal Medicine, CHA Bundang Medical Center, CHA University School of Medicine, Seongnam 13496, Republic of Korea

^2^Sungkyunkwan University School of Medicine, Seoul, Republic of Korea

^3^Department of Medicine, Samsung Medical Center, Sungkyunkwan University School of Medicine, Seoul, Republic of Korea

^4^Data Science Team, Hanmi Pharm. Co. Ltd., Seoul, Republic of Korea

^5^Department of Clinical Research Design and Evaluation, Samsung Advanced Institute for

Health Sciences and Technology, Seoul, Republic of Korea

**^†^**These two authors contributed equally.

**^*^Correspondence:**

Jae Hyeon Kim, MD, PhD

Department of Medicine, Samsung Medical Center, Sungkyunkwan University School of Medicine

81, Irwon-ro, Gangnam-gu, Seoul, 06351, Republic of Korea

Email: jaehyeon@skku.edu, Phone: +82-2-3410-1580

|  | **Events** | **HR (95% CI), p-value** | | | |
| --- | --- | --- | --- | --- | --- |
|  |  | **Model 1** | **Model 2** | **Model 3** | **Model 4** |
| **iHF** |  |  |  |  |  |
| Non-NAFLD | 4,639 | 1 (ref.) | 1 (ref.) | 1 (ref.) | 1 (ref.) |
| Regressed NAFLD | 208 | 1.528 (1.330-1.757), <0.001 | 1.127 (0.977-1.301), 0.101 | 1.110 (0.962-1.281), 0.154 | 0.960 (0.832-1.1080), 0.579 |
| Incident NAFLD | 161 | 1.525 (1.303-1.786), <0.001 | 1.427 (1.215-1.676), <0.001 | 1.389 (1.182-1.631), <0.001 | 1.197 (1.019-1.406), 0.029 |
| Persistent NAFLD | 453 | 1.900 (1.725-2.092), <0.001 | 1.705 (1.526-1.906), <0.001 | 1.643 (1.469-1.838), <0.001 | 1.363 (1.219-1.525), <0.001 |
| Persistent NAFLD | 453 | 1 (ref.) | 1 (ref.) | 1 (ref.) | 1 (ref.) |
| Regressed NAFLD | 208 | 0.801 (0.679-0.944), 0.008 | 0.668 (0.561-0.794), <0.001 | 0.665 (0.559-0.792), <0.001 | 0.710 (0.596-0.846), <0.001 |
| **hHF** |  |  |  |  |  |
| Non-NAFLD | 1,945 | 1 (ref.) | 1 (ref.) | 1 (ref.) | 1 (ref.) |
| Regressed NAFLD | 85 | 1.592 (1.281-1.979), <0.001 | 1.288 (1.030-1.611), 0.026 | 1.249 (0.999-1.562), 0.052 | 1.103 (0.882-1.380), 0.391 |
| Incident NAFLD | 51 | 1.318 (0.997-1.742), 0.052 | 1.409 (1.062-1.870), 0.018 | 1.328 (1.000-1.763), 0.050 | 1.197 (0.902-1.590), 0.213 |
| Persistent NAFLD | 187 | 1.982 (1.705-2.303), <0.001 | 2.222 (1.870-2.640), <0.001 | 2.059 (1.731-2.450), <0.001 | 1.780 (1.495-2.119), <0.001 |
| Persistent NAFLD | 187 | 1 (ref.) | 1 (ref.) | 1 (ref.) | 1 (ref.) |
| Regressed NAFLD | 85 | 0.802 (0.621-1.036), 0.092 | 0.571 (0.435-0.750), <0.001 | 0.586 (0.446-0.770), <0.001 | 0.604 (0.459-0.794), <0.001 |
| **All-cause mortality** |  |  |  |  |  |
| Non-NAFLD | 3,750 | 1 (ref.) | 1 (ref.) | 1 (ref.) | 1 (ref.) |
| Regressed NAFLD | 197 | 1.811 (1.569-2.091), <0.001 | 1.662 (1.433-1.927), <0.001 | 1.599 (1.379-1.855), <0.001 | 1.495 (1.288-1.735), <0.001 |
| Incident NAFLD | 132 | 1.599 (1.344-1.903), <0.001 | 1.917 (1.605-2.289), <0.001 | 1.764 (1.477-2.108), <0.001 | 1.678 (1.404-2.005), <0.001 |
| Persistent NAFLD | 253 | 1.317 (1.160-1.496), <0.001 | 1.873 (1.629-2.154), <0.001 | 1.693 (1.471-1.949), <0.001 | 1.561 (1.355-1.798), <0.001 |
| Persistent NAFLD | 253 | 1 (ref.) | 1 (ref.) | 1 (ref.) | 1 (ref.) |
| Regressed NAFLD | 197 | 1.375 (1.141-1.657), <0.001 | 0.941 (0.772-1.146), 0.545 | 0.989 (0.811-1.206), 0.911 | 1.001 (0.820-1.222), 0.989 |
| **CV mortality** |  |  |  |  |  |
| Non-NAFLD | 528 | 1 (ref.) | 1 (ref.) | 1 (ref.) | 1 (ref.) |
| Regressed NAFLD | 25 | 1.608 (1.076-2.403), 0.020 | 1.460 (0.966-2.206), 0.073 | 1.391 (0.919-2.104), 0.118 | 1.239 (0.819-1.876), 0.311 |
| Incident NAFLD | 27 | 2.253 (1.528-3.323), <0.001 | 2.724 (1.828-4.059), <0.001 | 2.463 (1.652-3.672), <0.001 | 2.215 (1.485-3.303), <0.001 |
| Persistent NAFLD | 38 | 1.391 (1.000-1.934), 0.050 | 2.012 (1.401-2.891), <0.001 | 1.769 (1.228-2.549), 0.002 | 1.543 (1.070-2.224), 0.020 |
| Persistent NAFLD | 38 | 1 (ref.) | 1 (ref.) | 1 (ref.) | 1 (ref.) |
| Regressed NAFLD | 25 | 1.156 (0.698-1.915), 0.574 | 0.866 (0.506-1.483), 0.601 | 0.880 (0.513-1.509), 0.642 | 0.877 (0.510-1.506), 0.633 |
| **Liver-related mortality** |  |  |  |  |  |
| Non-NAFLD | 131 | 1 (ref.) | 1 (ref.) | 1 (ref.) | 1 (ref.) |
| Regressed NAFLD | 10 | 2.561 (1.345-4.876), 0.004 | 2.746 (1.405-5.367), 0.003 | 2.528 (1.291-4.949), 0.007 | 2.456 (1.250-4.823), 0.009 |
| Incident NAFLD | 11 | 3.611 (1.945-6.707), <0.001 | 4.596 (2.417-8.737), <0.001 | 3.952 (2.076-7.523), <0.001 | 3.859 (2.021-7.371), <0.001 |
| Persistent NAFLD | 24 | 3.482 (2.251-5.387), <0.001 | 5.681 (3.382-9.543), <0.001 | 4.669 (2.757-7.906), <0.001 | 4.482 (2.626-7.648), <0.001 |
| Persistent NAFLD | 24 | 1 (ref.) | 1 (ref.) | 1 (ref.) | 1 (ref.) |
| Regressed NAFLD | 10 | 0.738 (0.353-1.543),0.419 | 0.443 (0.206-0.954), 0.037 | 0.494 (0.228-1.069), 0.074 | 0.492 (0.227-1.069), 0.073 |

**Table S1. Sensitivity analysis**^*^

Model 1: Crude.

Model 2: Age, sex, and body weight.

Model 3: Model 2 + alcohol consumption, smoking, regular exercise, and income status.

Model 4: Model 3 + hypertension, diabetes mellitus, dyslipidemia, and estimated glomerular filtration rate.

CI, confidence interval; CV, cardiovascular; hHF, hospitalized heart failure; HR, hazard ratio; iHF, incident heart failure; NAFLD, non-alcoholic fatty liver disease

^*^Subjects whose FLI categories were subsequently changed after the first two years were censored for sensitivity analysis.
